# Bacteria Halotolerant from Karst Sinkholes as a Source of Biosurfactants and Bioemulsifiers

**DOI:** 10.3390/microorganisms10071264

**Published:** 2022-06-21

**Authors:** Félix Maldonado Desena, Navila De la Cruz Ceferino, Sergio Gómez Cornelio, Carina Alvarez Villagomez, José Luis Herrera Candelario, Susana De la Rosa García

**Affiliations:** 1 División Académica de Ciencias Biológicas, Universidad Juárez Autónoma de Tabasco, Carr. Villahermosa-Cardenas Km 0.5, Ranchería Emialiano Zapata, Villahermosa 86150, Tabasco, Mexico; fello.oficial15@gmail.com (F.M.D.); delacruznavila2329@gmail.com (N.D.l.C.C.); carina.alvarez@ujat.mx (C.A.V.); luisherrera504@gmail.com (J.L.H.C.); 2 Ingeniería en Biotecnología, Universidad Politécnica del Centro, Carretera Federal, Villahermosa-Teapa Km 22.5, Tumbulushal Centro, Villahermosa 86290, Tabasco, Mexico; sagomezcornelio@gmail.com

**Keywords:** biofilms, sessile culture, sinkholes, halotolerance, bioremediation, optimization

## Abstract

Halotolerant bacteria with biosurfactant (BS) and bioemulsifiers (BE) activity can coexist in Karstic sinkholes with marine influence. Two sinkholes in the Yucatan peninsula were selected to isolate bacteria with BE and BS activity stable in NaCl. The optimal time, the effect of nitrogen and carbon source in the medium, and the conditions (agitation, pH and salinity) for the production of BS and BE compounds in planktonic and sessile (stimulate the formation of biofilms in cell roller) culture were determined. Eighty strains showed the highest emulsification activity (EI_24_ ≥ 50%) and drop-collapse ≥ 4 mm. 87% of the strains are moderately halotolerant, and 21% bordered the limit of extreme halotolerance. Twenty-four strains maintained or improved their BS and BE activity under salinity conditions at 5% and 10%, being the most active genera *Bacillus*, *Paenibacillus* and *Lysinibacillus*, identified by sequencing of the 16S rRNA gene. The results show that the nitrogen source positively affects the BS and BE activity, regardless of the type of culture. The sessile culture markedly stimulated BS activity with significant differences. However, we did not find a greater influence on the culture conditions. The results suggest that halotolerant bacteria from sinkholes could be implemented in bioremediation and other biotechnological applications.

## 1. Introduction

The sinkholes (cenotes) that predominate in the Yucatan Peninsula originated from a geomorphological process called karst, resulting from the dissolution of karst rock with permeability through a low hydraulic gradient, which causes the filtration and accumulation of water, generating a layer of fresh water floating on a mass of saline water, whose principle is the intrusion of seawater [1,2]. The oligotrophic and alkaline conditions of the sinkholes are due to the contribution of carbonates and bicarbonates from groundwater [3], which, with the halocline environment, give rise to a little-explored autochthonous microbial community capable of growing and tolerating high concentrations of chloride ions [4]. In these environments, chemoautotrophic bacteria associated with sediments and submerged walls predominate [5], and in sinkholes with low or no anthropogenic impact (pristine), cultivable populations of Gram-negative bacteria of the genera *Pseudomonas*, *Burkholderia*, *Sphingomonas* and *Bacillus* have been reported, including strains of the genus *Photobacterium* of marine origin [6].

Studies on the potential of the microorganisms that inhabit the sinkholes are scarce. Bacteria that produce secondary metabolites, such as bacteriocins, siderophores and biosurfactant compounds with antimicrobial activity have been found [6]. The sinkholes of halotolerant, oligotrophic and alkaline conditions may be of interest in the search for metabolites with biotechnological applications, especially to remediate sites contaminated by hydrocarbons, whose salinity, osmolarity, temperatures and pH conditions are altered, affecting ecosystems and the food chain [7,8].

A variety of microorganisms, such as bacteria, fungi and yeasts, produce biosurfactants (BS), reducing the surface tension between two phases with diverse and low molecular weight compounds, such as glycolipids, polypeptides and lipopeptides [9]. In contrast, bioemulsifiers (BE) that are high molecular weight compounds made up of polysaccharides, lipopolysaccharides and lipoproteins can produce and stabilize emulsifications for a long time [10]. These represent an important alternative to synthetic surfactants [11,12], which are not very effective and are friendly to the environment since they release other types of chemical compounds that, due to their persistence, cause harmful side effects to the environment [13,14].

The nature and performance of BS and BE compounds will depend on the type of microorganism, medium composition and culture conditions in which they are produced. These compounds for bioremediation processes must be produced by innocuous microorganisms, with efficiency in short times and economic culture media [15], low ecotoxicity, and high surface-active and emulsifying activity under conditions environmental extremes (pH, temperature, and salinity). Microorganisms used in bioremediation processes are frequently isolated from contaminated soils or wastewater effluents [16,17]; however, undisturbed sinkholes could be an interesting alternative since the adaptation and survival strategy, particularly under variable conditions, would contribute to the production of BS and BE [13,18], allowing greater bioavailability on various hydrophobic substrates, favouring adherence and therefore the establishment of biofilms. Halotolerant bacteria are a clear example of adaptation to stress conditions since they have various strategies that allow them to withstand wide salinity ranges [19], such as those that prevail in the sinkholes of the Yucatan peninsula.

The microbial communities that proliferate under the particular conditions of the sinkholes of the Yucatan Peninsula may be of interest for various biotechnological applications; in this work, bacteria from two sinkholes with little anthropogenic impact were isolated, and those that produce biosurfactant compounds and stable bioemulsifiers in salinity conditions were selected. For the five bacteria with the highest activity, the time of highest BS and BE activity were determined in planktonic (shaken flask) and sessile (the stimulation of biofilms formation in a rolling bottle system) cultures. After the the time of greatest activity BE (EI_24_) and BS (drop-collapse) was determined, the composition of the medium (complex carbon and nitrogen sources) and culture conditions (pH, salinity and agitation) were optimized to establish the variables that stimulate the biosynthetic capacity of secondary metabolites with BS and BE activity. These results make it possible to contribute to the large-scale production of biosurfactants under a cultivation system that stimulates the expression and is economically profitable for its potential use in bioremediation, pharmacy, food and biological control, among others.

## 2. Materials and Methods

### 2.1. Study Site and Sampling

The sinkholes were selected for easy access and with the least anthropogenic influence that could alter the microbiota. These are located in the archaeo-ecological reserve of Dzibilchaltún at approximately 30 km from the Gulf of Mexico, named by locals as Temozón (21°04′23″ N and 89°36′00″ W) and X′ can ho che (21°04′17″ N and 89°36′26″ W). Samples of water, sediment and wall scraping were collected and transported cold to the laboratory.

### 2.2. Isolation of Bacteria

Due to the oligotrophic conditions reported for the cenotes of the Yucatan Peninsula, the bacteria were isolated in a trypticase soy agar (TSA) medium diluted to 10% with sinkhole water filtered by a 0.45 μm Millipore membrane and 13.5 g/L of agar were added [6]. From the water samples, 60 μL were taken and inoculated in the culture media; on the other hand, 1 g of each sediment and wall scraping samples were suspended in 9 mL sterile water from the sinkhole. The suspensions were serially diluted (10^−1^ to 10^−4^); 60 μL of each dilution was plated and incubated at 35 °C. Colonies with different morphology were transferred to plates with TSA until pure cultures were obtained (Figure S1a). The purity of the isolated was verified by Gram staining and preserved in sterile water and deep-frozen (−80 °C) in trypticase soy broth (TSB) with 25% glycerol added [20].

### 2.3. Selection of Strains with Biosurfactant (BS) and Bioemulsifying (BE) Activity

For selecting strains with BS and BE activity, 10 mL of the overnight cultures adjusted to an optical density (OD_550nm_) of 0.13, which corresponds to 1.5 × 10^8^ colony-forming units (CFU), were inoculated in 90 mL of TSB and incubated at 150 rpm, 35 °C for 72 h. After they were centrifuged (5350× *g*, 30 min, 4 °C), the cell-free supernatants (CFS) were used for the quantification of the emulsifying capacity and the biosurfactant activity.

#### 2.3.1. Emulsifying Activity (EI_24_)

In test tubes for triplicate, 2 mL of the CFS of each bacterium and 2 mL of *n*-hexadecane were added and vortexed at 200 rpm for 2 min [21]. The emulsion was left to stand for 24 h in the dark to measure the stable area of emulsification. The emulsification index was defined as the height of the emulsification divided by the total height, expressed as a percentage, and Equation (1) [22] was applied. As a positive control, the commercial surfactants Triton^TM^ X-100 (Merck, Darmstadt, Germany) and Tween 80 (non-ionic surfactants), sodium dodecyl sulphate (SDS) (anionic surfactant) at 1%, were used. Non-inoculated TSB medium was used as negative control.

(1) $${\mathrm{EI}}_{24}=\frac{\mathrm{Height}\;\mathrm{of}\;\mathrm{the}\;\mathrm{emulsion}\;\mathrm{layer}\;\left(\mathrm{mm}\right)}{\mathrm{Total}\;\mathrm{height}\;\mathrm{of}\;\mathrm{the}\;\mathrm{liquid}\;\mathrm{column}\;\left(\mathrm{mm}\right)}\times 100$$

The strains with an emulsification index ≥50% were selected. In addition, these emulsions were kept in the dark to evaluate their stability one month and one year later.

#### 2.3.2. Biosurfactant Activity (Drop-Collapse)

In 96-well microplate covers, 2 µL of mineral oil was deposited in each well and stabilized for 24 h on a level surface. Subsequently, 5 µL of the CFSs were placed on the oil monolayer and one minute after application, the size and morphology of the droplet were inspected. Each CFS was evaluated in octuplicate and reported in mm [23]. The strains that caused the collapse of flattened droplets with sizes ≥4 mm were selected. As a positive control, commercial surfactants Triton^TM^ X-100, Tween 80 and SDS were used at their corresponding critical micelle concentration (CMC) for each surfactant.

#### 2.3.3. Stability of BS and BE Activity under Salinity Conditions

The CFS from each strain that presented good emulsifying and surfactant activity, NaCl was added to a final concentration of 5% and 10%, and the emulsifying and biosurfactant activity was re-evaluated. The commercial surfactants Triton^TM^ X-100, Tween 80 and SDS at 1% final concentration were used as a positive control and the TSB medium was used as negative control, and 5% and 10% NaCl were added.

#### 2.3.4. Halotolerance Test

The isolates were cultivated in a salinity gradient of 2.5%, 5%, 7.5%, 10%, 12.5%, 15%, 17.5% and 20% NaCl using TSA as the base medium [23]; and only TSA medium (0.5% NaCl) as control (Figure S1b).

#### 2.3.5. Haemolytic Activity

The blood–agar base medium was sterilized; at about 50 °C, defibrinated blood was added at 5% (*v*/*v*) and gently shaken. Subsequently, the blood–agar medium was poured into Petri dishes of 10 × 10 cm, equidistantly, containing 16 metal cylinders, which were removed when the agar gelled. 100 µL of CFS of each strain were placed in the wells, Triton^TM^ X-100 at 1% was used as a positive control, and sterile TSB was used as a negative control. The plates were incubated at 25 °C for 24 h; the diameter of haemolytic halos was evaluated in mm, recording the colour changes around the well [24].

### 2.4. Motility Test: Swimming and Swarming

The swimming and swarming motility of the selected strains were determined by applying 100 μL of the bacterial suspensions (1.5 × 10^8^ CFU) in a Luria–Bertani medium supplemented with 0.3% bacteriological agar for swimming and 0.7% for swarming, at 35 °C [25,26]. Luria–Bertani medium with 1.5% agar was used as growth control. The motility was recorded as the diameter (cm) of displacement from the point of inoculation at 24 and 48 h.

### 2.5. Molecular Identification of Bacteria with BS and BE Activity Stable in Salt

Strains with higher BS and BE activity and stability under salinity conditions were identified by amplifying 16S rRNA using primers fD1: AGAGTTT-GATCCTGGCTCAG and rD1: AAGGAGGTGATCCAGCC by PCR [27]. The purified DNA fragments were sent for sequencing. The taxonomic location of the bacterial strains was performed by homology search by comparison with the nucleotide sequences of the 16S rRNA gene reported in the GeneBank (https://www.ncbi.nlm.nih.gov/genbank/; accessed on 15 May 2022) of the National Center for Biotechnology Information (NCBI), using the BLAST sequence analysis tool (http://www.ncbi.nlm.nih.gov/BLAST/; accessed on 15 May 2022). The sequences were deposited in the Genbank database.

### 2.6. BS and BE Production at Different Times (h) in Planktonic Culture

The selected strains *B*. *vallismortis* XHA16, *B*. *siamensis* XHA14, *Bacillus* sp. TZS01, *L*. *fusiformis* TZA38 and *Paenibacillus* sp. XHA18 were grown at 35 °C in TSB. Overnight bacterial cultures were adjusted to OD_550nm_ of 0.13. 100 mL of this culture were added to 900 mL of TSB and incubated at 120 rpm, pH 7 for 96 h. Monitoring in the time of BE and BS activity was performed by taking 35 mL aliquots in triplicate every 24 h up to 96 h, each sample was centrifuged to obtain the CFS, and BE and BS activity was evaluated, as described previously (Section 2.3.1 and Section 2.3.2).

### 2.7. BS and BE Production at Different Times (h) in Sessile Culture

For sessile culture, 18 mL of TSA at 1.6% were deposited in 100 mL Duran bottles. The bottles were sterilized and then rotated on ice for 5 min to coat the entire internal surface. Overnight bacterial cultures were adjusted to OD_550nm_ of 0.13 and added to the bottles and spread by rotation. The bottles were incubated in a rotary culture system at 7 rotations per minute (rpm) at 35 °C for 24 h, and then 10 mL of fresh TSB culture medium was added [28]. Every 24 h, two bottles were removed to determine the BS and BE activity, up to 96 h, as described in numbers (Section 2.3.1 and Section 2.3.2); in addition, stability at 5 and 10% NaCl was evaluated at the time (h) of the highest BS and BE activity [29].

### 2.8. Optimization of the Composition of the Culture Medium for BS and BE Activity

The composition of the carbon source for the culture medium was molasses, and yeast extract was the nitrogen source; both represent the fundamental components for the production of BS by bacteria of the Bacillaceae family [30,31]. To determine the appropriate combinations for the production of BS and BE, a 3^2^ factorial design was implemented; F1 corresponds to molasses with three levels 1%, 2% and 3%, and F2 to yeast extract at 0.55%, 1.25% and 2% in planktonic and sessile cultures (Table 1). The assays were performed at the times of highest expression of BS and BE activity (Section 2.6 and Section 2.7), at 7 rpm in sessile and 150 rpm in planktonic cultures at 35 °C. The cultures were centrifuged (5350× *g*, 30 min, 4 °C), and the pH of the CFSs were determined. BS and BE activity were evaluated as described above (Section 2.3.1 and Section 2.3.2) with the replacement of *n*-hexadecane by diesel in BE activity.

### 2.9. Optimization of Sessile and Planktonic Culture Conditions for BS and BE Activity

The pH condition was determined in a pH range of 4 to 10. 100 µL overnight culture adjusted to OD_550nm_ of 0.13 was added to tubes with 9.9 mL of TSB and shaken at 150 rpm for 48 h at 35 °C. Microbial growth was determined at OD_550nm_. Based on these results, a fractional factorial design of the culture conditions was carried out, with pH values of 6, 7 and 8; salt concentrations at 0.5%, 2.5% and 4.5% and agitation of 4, 7 and 10 rpm in the sessile culture and 120, 150 and 180 rpm in planktonic culture (Table 1). The cultures were then centrifuged (5350× *g*, 30 min, 4 °C), and the final pH of the CFSs were determined. BS and BE activity were evaluated as described above (Section 2.3.1 and Section 2.3.2), replacing *n*-hexadecane with diesel.

### 2.10. Statistical Analysis

The BS and BE activity results were analysed by multiple comparisons of means, performing a one-way analysis of variance (ANOVA), followed by a Tukey test at a significance of *p* < 0.05. In addition, to optimise the medium and the culture conditions, the response surface method and principal component analysis with the Statistic 7.0 software (Statistic, Stat Soft, Inc, Tulsa, OK, USA).

## 3. Results and Discussion

### 3.1. Isolation of Cultivable Bacteria from Sinkhole

The use of a culture medium that mimics the natural environment conditions is essential for the recovery of a greater diversity of microorganisms. From both sinkholes, 170 different strains were isolated. The 109 isolates from the sinkhole X′ can ho che (XH) were recovered from the water samples; from Temozón (TZ), 61 strains were isolated, 5 from wall scrapings, 3 from sediment and 53 from water. The recovery of microorganisms from oligotrophic environments is generally low since less than 10% can be cultivated [32], particularly in undisturbed environments [18].

The low anthropogenic impact of these sinkholes supposes the recovery of native microorganisms since the bacteria isolated from these sinkholes are sensitive to 400 and 500 ppm of heavy metals nickel, copper, lead and mercury. In addition, the low concentration of nitrates in the water is a good indicator of minimal human activity and the incorporation of fertilizers [33].

### 3.2. Biosurfactant and Bioemulsifying Activity of Strains Isolated from Sinkholes

**Figure 1:**
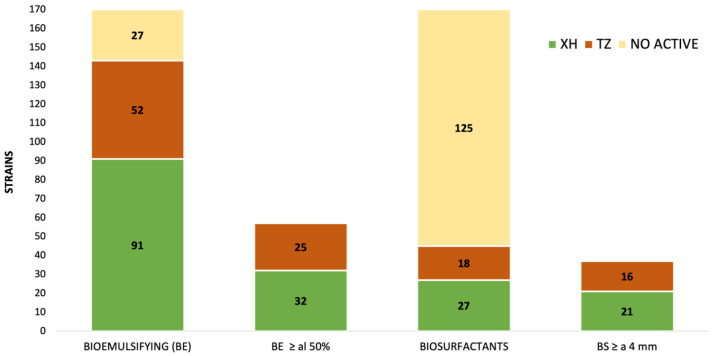
Bioemulsifying (BS) and biosurfactant (BS) activity, by strains isolated from the X′ can ho che (XH) and Temozon (TZ) sinkholes and their highlighting the EI_24_ ≥ 50% and drop-collapse ≥ 4 mm.

The 170 strains were evaluated for their emulsifying activity with the *n*-hexadecane at 24 h and their biosurfactant activity by drop-collapse assay. Most strains isolated from the sinkholes (84%) were able to produce metabolites with BE activity and only 26% with BS activity (Figure 1).

BE activity was observed with a similar percentage of bacteria in both sinkholes, TZ with 85% and XH with 83% (Figure 1); of these, 33.5% produce EI_24_ ≥ 50%. This suggests that the cultivable microorganisms from these sites can produce metabolites with BE activity, which may be a mechanism to efficiently solubilize low nutrients in this oligotrophic environment [18].

Regarding the BS activity, according to Joy et al. [34], good activity can occur depending on the flattening of the droplet; therefore, those strains with a size ≥4 mm were selected. BS activity in the sinkholes was 29% and 24% for the strains isolated from TZ and XH, respectively (Figure 1). This highlights the higher percentage (33.5%) of the recovered strains with the capacity of producing emulsifications ≥50% and a minor percentage (26%) with surface-active activity (drop-collapse ≥ 4 mm). However, strains producing biosurfactants with the bioemulsifying compound have been documented, but not vice versa, and there is even a strong negative correlation between the measurement of surface and the interfacial tension. Therefore, the BS activity shows the changes in the surface tension, while the BE shows activity in the stability of the emulsification–emulsion systems [35].

### 3.3. Stability of the BS and BE Activity under Salinity Conditions

The 80 strains with emulsifications (EI_24_) ≥50% and/or with a surface tension by the drop-collapse ≥ 4 mm, were evaluated at concentrations of 1% to 20% NaCl (Table S1), considering halotolerant those that grow at concentrations above 1% of salt [36]; therefore 94% are halotolerant and only 4% do not tolerate salt. Most active strains grow between 2.5% and 20%, classifying them as moderately halotolerant, more frequent than those growing at 10% NaCl, while 21% border on extreme halotolerant [19,37]. The ability to tolerate salt concentrations by these bacteria from freshwater sinkholes is attributed to their halocline environment [3]. It has also been shown that microorganisms and crustaceans from these sites tolerate moderate salt concentrations, finding some species of marine origin [5,6,38].

The stability of the metabolites responsible for the BS and BE activity under two salinity concentrations were determined. Of the 32 strains from sinkhole XH that presented an EI_24_ ≥ 50% with *n*-hexadecane (Table S1), only 17 maintained stable the EI_24_ at 5% and 10% NaCl; however, in the remaining strains, the loss of emulsification was not greater than 40% (Table S1). Despite the dominance of Gram-negative bacteria in the sinkholes, the Gram-positive presented the most stable emulsifying activities in salinity conditions. In addition, of the 25 strains with EI_24_ ≥ 50% in the TZ sinkhole, 76% and 72%, maintain emulsifying stability at 5% and 10% NaCl, respectively.

Regarding BS activity, of the 21 strains from the XH sinkhole with biosurfactant activity ≥4 mm, 10 maintained their activity at 5% and seven strains at 10% NaCl. Similar to the BE activity results, Gram-positive bacteria expressed metabolites of a more stable nature under salinity conditions. Similar results were observed in TZ, with better BS activity in 14 and 13 strains at 5% and 10% salt concentrations, respectively; predominantly Gram-negative strains, in contrast to Gram-positive from sinkhole XH (Table S1). This difference in the BS activity and the dominance of a bacterial group in the TZ sinkhole is likely due to the competition for the scarcity of organic matter so that bacteria with the best capacity to sequester and assimilate nutrients will be the most successful [18].

In response to environmental stress, such as salinity, microorganisms trigger the biosynthesis of various metabolic compounds to counteract the effects [39]. In this sense, halotolerant bacteria implement a regulation mechanism by synthesizing and/or accumulating compatible solutes within the cell to maintain the osmotic balance; these osmoregulation mechanisms are used to colonize saline environments [40,41]. Generally, halotolerant bacteria belong to genera, such as *Pseudomonas, Flavobacterium, Staphylococcus, Acinetobacter, Vibrio, Micrococcus, Alteromonas*, and *Bacillus* [42]. Similar genera, such as *Pseudomonas, Staphylococcus*, and *Bacillus* were found in this study.

The contaminated sites are characterized by high salinity gradients, making their bioremediation difficult by conventional methods. However, the microorganisms that are not halotolerant and used to treat contaminated water and soil can suffer cell lysis since the salt denatures enzymes and dehydrates cells [43]. Therefore, to remedy these sites and marine environments, it was necessary to use halophilic or halotolerant microorganisms that withstand saline environment conditions [44]. Therefore, the high number of halotolerant strains from cenotes and their capacity to produce emulsions (BE) and break the surface tension (BS) at 5 and 10% salt concentration is transcendental.

### 3.4. Haemolytic Activity of Strains with Stable BS and BE Compound under Salinity Conditions

Haemolytic activity is related to BS activity, and the haemolysis assays are utilized as a quick way to select strains that produce metabolites with BS activity [23]. The rupture of the erythrocyte membrane is due to hydrophilic and hydrophobic nature of the metabolites [45]. Haemolytic activity was evaluated from the CFS obtained at 72 h on 5% blood–agar plates. The results show that 49 of the 80 strains evaluated can break the surface tension with a drop-collapse ≥4 mm and only 23 presented haemolytic activity (Table 2 and Table S1). The largest haemolysis halo was recorded by the XHA02 bacterium (2.7 mm); however, it did not show a notable BS activity (>3 mm) (Table S1). According to other studies, there is no 100% relationship between bacteria with BS activity and those with haemolytic activity, since metabolites that break surface tension do not always cause the haemolysis of erythrocytes. Therefore, the compounds responsible for the haemolytic activity are not necessarily of the biosurfactant type, such as the haemolysin-type exotoxins [46].

### 3.5. Motility Test: Swimming and Swarming

Previous studies have shown a significant association between the motility mechanisms of bacteria and the BS production, finding strains deficient in BS compounds that do not form swarming, nor do they manage to form biofilm [25]. In the motility tests, it was found that the size of the colony of *B. siamensis*, *B*. *vallismortis*, *Bacillus* sp. and *L*. *fusiformis* spread on the surface of the plate (8.5 cm) from the first 24 h of swimming and swarming with a waxy biofilm, except for *L. fusiformis*, which presented a type of mucous biofilm. On the other hand, the motility of *Paenibacillus* sp. presented a circular colony margin and mucosal biofilm with 1.15 cm for swimming at 48 h and swarming with 1.25 cm. Various studies show that lipopeptide-type BS affects bacteria’s growth, helping to assemble multicellular communities [26]. Lipopeptides, such as surfactin and mycosubtilin, contribute to the extension of the bacteria on the surface and decrease surface tension [47], so the swarming activity of strains from sinkholes may be mediated by the production of lipopeptide-type BS.

### 3.6. Molecular Identification of Strains with Stable BS and BE Activity in Salinity Conditions

The 24 most promising strains for their stable BE and/or BS activity in salinity conditions were identified by gene 16 rRNA sequencing (Table 2 and Figure S2). A variety of genera coincided with those reported as producers of metabolites with BS activity, *Pseudomonas*, *Bacillus* and related genera, such as *Paenibacillus*, *Brevibacillus* and *Lysinibacillus*. In this sense, few studies have reported species of the *Brevibacillus* genus as producers of BS metabolites with EI_24_ ≥ 50% [48], except under optimized conditions that have an EI_24_ of 72% [49].

Species identified by gene 16 rRNA, such as *B. siamensis*, have been associated with BE activity and antifungal activity [50] as plant growth promoters [51] and as bioremediators in salinity conditions [52]. The *Bacillus* and *Psedomonas* genera are known for their versatility and ability to colonize different environments and use various substrates due to the diversity of enzymes and metabolites produced, so these genera are highly explored for biotechnological applications. *Bacillus sphearicus* and *B*. *fusiformis* are now relocated to the genus *Lysinibacillus* for their typical spherical or ellipsoidal endospores [53].

The *Lysinibacillus* has potential by degrading heavy metals, producing substances that promote plant growth and compounding bioinsecticide [53]. While *L. fusiformis* isolated from soil contaminated by automobile-mechanic workshops has a notable production of BE metabolites, with EI_24_ of 65.15% and optimized conditions, it increased to 93.08% [54].

The origin of the bacteria isolated in this study demonstrates the influence of the marine environment on the sinkholes, as is the case of *B. vallismortis*, which is a strain of marine origin and considered extremophilic [55], with emulsifications greater than 50%; while in this study they were greater than 60%. In addition, their BS activity is attributed to a lipopeptide of the type surfactin and iturin A [56].

### 3.7. Expression at Different Times of Metabolites with BS and BE Activity in Planktonic and Sessile Cultures

**Figure 2:**
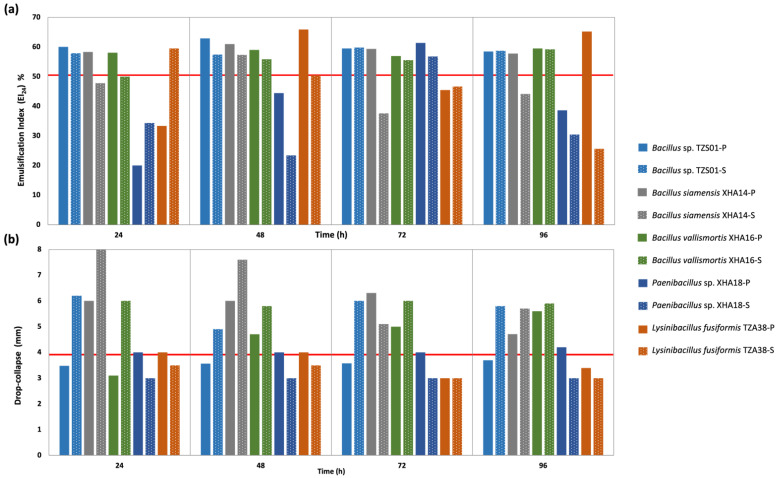
(**a**) Bioemulsifying and (**b**) biosurfactant activity of bacteria strains from sinkholes at different times in planktonic and sessile cultures. Solid bars are planktonic culture (P), dotted bars are sessile culture (S).

The bacteria *B*. *vallismortis* XHA16, *B*. *siamensis* XHA14, *Bacillus* sp. TZS01, *L*. *fusiformis* TZA38 and *Paenibacillus* sp. XHA18 with stable BE and/or BS activity in salinity culture were selected (Table 2) to determine the time of greatest expression of these compounds in planktonic and sessile cultures. In sessile, BE activity with EI_24_ higher than 59% in diesel substrate between 24 and 96 h was recorded, highlighting *L*. *fusiformis* at 24 h, *Bacillus* sp. at 72 h and *B*. *vallismortis* at 96 h (Figure 2a). No significant differences in the production of BE compounds between both cultures were found (*p* < 0.05). Only *B*. *vallismortis*, *B*. *siamensis* and *Bacillus* sp. showed stable emulsifications without significant differences in the times evaluated in the sessile culture. *Paenibacillus* sp. presented the highest EI_24_ at 72 h, *L*. *fusiformis* at 48 and 96 h and *Bacillus* sp. at 48 h (EI_24_ greater than 60%) in the planktonic culture (Figure 2a). In most cases, the bacteria produced BE compounds at different times (h); in contrast, most studies state that the BE compounds are produced at the end of the exponential phase or the beginning of the stationary [57,58,59].

Statistically significant differences were observed in BS activity between the sessile and planktonic cultures (*p* < 0.05) (Figure 2b). In the planktonic, the highest activity was recorded in four strains after 72 h, ranging from 3.58 *(Bacillus* sp.) to 6.31 mm (*B*. *siamensis*). While in sessile culture, *B*. *siamensis*, *B*. *vallismortis* and *Bacillus* sp. presented an activity ≥6 mm from the first 24 h (Figure 2b), contrasting with *L*. *fusiformis* and *Paenibacillus* sp., where the drop-collapse was less than 4 mm from 24 h. However, there was no relationship between BE compounds’ production and the BS activity in sessile culture. The production of BS compounds could contribute to improving the hydrophobicity of the cell surface from the first hours of growth since, in low salinity conditions, bacterial cells balance intra- and extracellular osmotic levels, promoting the expression of bioemulsifiers compounds, accelerating the formation of micelles and with it the increase in the availability of the hydrocarbon, managing to degrade those of long-chain [24].

Most of the values obtained in the EI_24_ are considerably high, from 24 h to 96 h of culture; this suggests that the types of BE produced by the bacteria at the different times evaluated change the modes of access to the substrate diesel [14,60,61]. In this case, the stability and formation of the emulsion are of importance; therefore, the expression of a surfactant-type compound contributes to the bioremediation process efficiently since the surfactant is absorbed in the interface between water and oil, which reduces the interfacial tension and prevents the coalescence of the droplets, increasing the repulsion forces between them [62]. In addition, high molecular weight bioemulsifiers tightly absorb the hydrocarbons and thus increase their apparent solubility [63].

The salt stability (5% and 10%) of each strain’s BE and BS metabolites at the best expression time in sessile culture was evaluated. In general, it was observed that both BS and BE activity decreased with increasing salt concentration with statistically significant differences (*p* < 0.05); however, in the EI_24_ of *Bacillus* sp. and *L*. *fusiformis* there are no significant differences between the BE activity without salt with the two salt concentrations. Results were similar to the species that presented BS activity, where the activity generally decreased with the increase in salt, except *B*. *vallismortis*, where the biosurfactant activity increased significantly at 5% NaCl, which could be due to its origin extremophile [55].

### 3.8. Optimization of the C/N Source in the Medium to Produce BS and BE Compounds in Sessile and Planktonic Cultures

**Figure 3:**
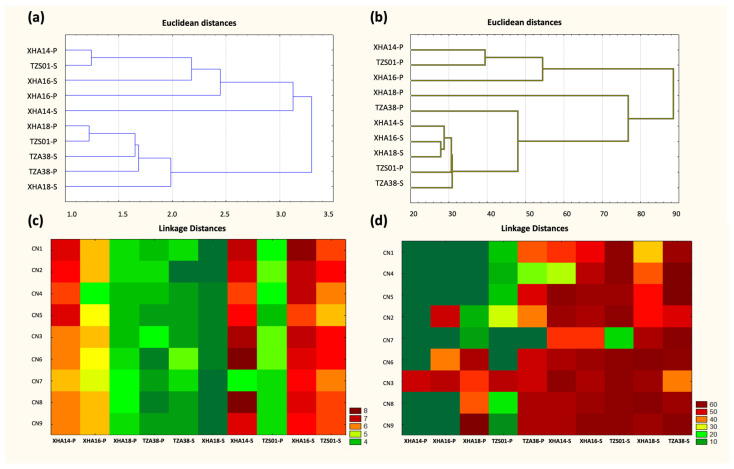
Euclidean distance of the BE (**a**) and BS (**b**) activity, and heat map EI_24_ (**c**) and drop-collapse (**d**) of halotolerant bacteria at different concentrations of C/N source in planktonic (P) and sessile (S) cultures.

The production and recovery of BS and BE compounds must be efficient and profitable for the industry and their respective biotechnological applications, since the commercial culture media that are frequently used increase production costs [64]. Therefore, the carbon source in the culture medium was replaced by molasses, an economical and easily accessible substrate for microorganisms, and the yeast extract as a nitrogen source, thereby determining the C/N combination and the culture type that influences a greater expression of BS and BE compounds. For BE activity, a positive correlation was found in principal component analysis (PCA) with the different nitrogen concentrations in both planktonic and sessile cultures (Table S2), except for *L. fusiformis* in the sessile culture (−0.426). While a negative correlation was found with the carbon source in most of the strains, according to the PCA and response surface analyses (RSA), only a positive effect in the BE production of *Paenibacillus* sp. in planktonic and sessile cultures was observed, and *L. fusiformis* in sessile culture at high carbon concentration according to RSA (Table S2 and Figure S3). In Euclidean distance analysis groups, the BE activity resulted in the five strains in the sessile culture agglomerating into a single cluster, while planktonic cultures differed in the distances between them (Figure 3a). Although the yeast extract can improve emulsification rates [65], it has been observed to cause a lowered ability to reduce surface tension [66]. However, these results show that the nitrogen source is relevant for producing BE and BS compounds (Table S2), finding that the BE activity at low nitrogen concentrations varies depending on the strain. Therefore, the sustained activity at different nitrogen concentrations may be due to the protein synthesis being blocked by shifting metabolism to carbohydrate synthesis, thus maintaining BS and BE activity [31].

In all the strains, the BE activity in planktonic culture was not recorded for some of the C/N ratios; only *L. fusiformis* showed activity for nine combinations of C/N with EI_24_ from 23% up to 55.9% (Figure 3c), except for the lowest concentration of nitrogen and the highest concentration of carbon (CN7); the RSA suggests that adding a higher carbon concentration would increase the BE activity for this strain (Figure S3). The BE activity was stable and consistent (EI_24_ of 40%) for all the species evaluated with the CN3 composition, the highest yeast extract concentration and low molasses concentration (Figure 3c), suggesting that bacteria efficiently metabolize the low concentration of molasses in planktonic culture. In the sessile culture, the effectiveness of the different carbon and nitrogen source concentrations stands out, with values greater than 35% of EI_24_ (Figure 3c), except for *Bacillus* sp. at low nitrogen concentration and high carbon (CN7). In general, no significant differences were observed in the different compositions in sessile culture; those with EI_24_ greater than 55% were the compositions that included 2% yeast extract and 3% molasses (CN9), and all the compositions of 3% molasses had concentrations greater than 1.25 yeast extract.

The biosurfactant activity measured through the drop-collapse in the optimization of the medium in sessile culture shows that all species of the genus *Bacillus* have a collapsed droplet size greater than 4.3 mm with statistically significant differences (*p* < 0.05) in *L. fusiformis* with a drop-collapse between 3 and 4.1 mm, and *Paenibacillus* sp. with values less than 3.5 mm in the different C/N combinations (Figure 3d). In planktonic culture, the largest droplet collapse was observed for *B. vallismortis*, *Bacillus* sp. and *L. fusiformis* in the composition CN3 (lowest carbon and highest nitrogen); while for *B. siamensis*, values of 6.56 mm were found in the minor and intermediate formulation of both sources of C/N (CN5) (Figure 3d). The PCA shows a positive correlation of most species to yeast extract in sessile and planktonic cultures (Table S2), except for *B. siamensis* and *Paenibacillus* sp. in the planktonic culture, which registers a negative correlation, and *B. vallismortis* in sessile culture according to the response surface (Figure S4). For molasses, most species correlation were negative, except for *Paenibacillus* sp. in planktonic culture and *L. fusiformis* in a sessile culture which presented a positive correlation. However, no correlation was observed between the two culture types in the BS activity similarity dendrogram (Figure 3b).

The pH of the CFS in planktonic and sessile cultures ranged between 5.49 and 8.76, highlighting that sessile culture with the composition of 0.55% yeast extract and 3% molasses (CN7) had the lowest values of pH for all strains (5.4 to 6.6), except *L. fusiformis*, which was 7.8. In the case of the planktonic culture, no significant differences were found for the strains *B. siamensis* and *L. fusiformis*, since the former had values from 6.3 to 7.2, while the latter had values from 7.7 to 8. The low variability in the pH of the CFSs suggests that the bacteria optimize the various conditions of the medium, maintaining the pH between 6 and 8, which could increase the biosynthesis of BS and BE [67,68,69].

### 3.9. Optimization of Culture Conditions for the Production of BS and BE Compounds in Sessile and Planktonic Cultures

**Figure 4:**
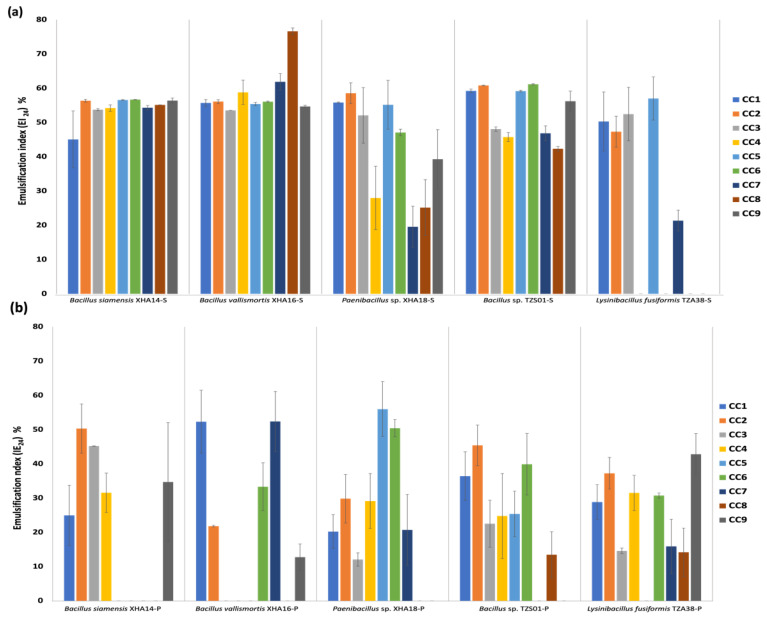
Optimization of culture conditions for bioemulsifier activity in sessile (**a**) and planktonic (**b**) systems of halotolerant bacteria.

**Figure 5:**
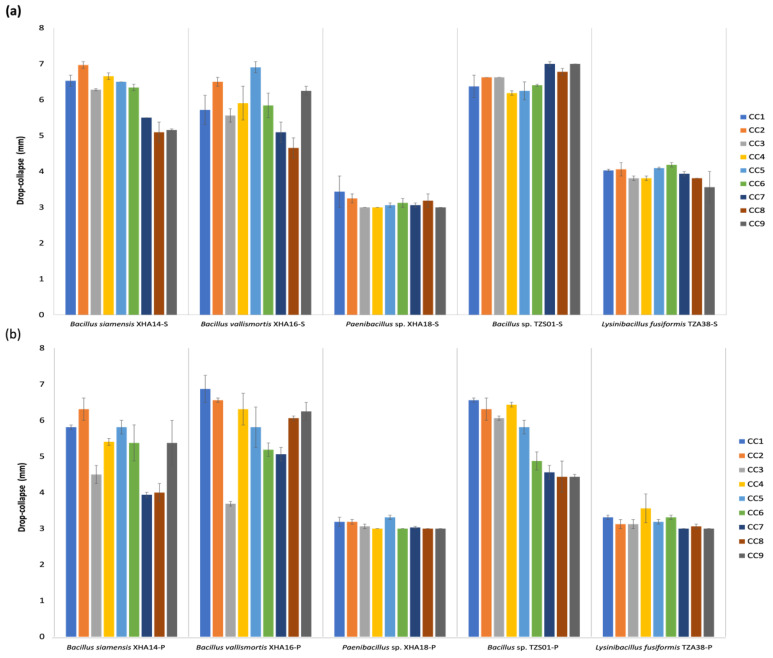
Optimization of culture conditions for biosurfactant activity, in sessile (**a**) and planktonic (**b**) systems of halotolerant bacteria.

The effects of agitation, pH and salt concentration to optimize the expression of BS and BE compounds in sessile and planktonic cultures were determined. The values obtained of the BS and BE activity in the culture conditions (Figure 4 and Figure 5) do not exceed the observed in the optimization of the medium (C/N) (Figure 3). Although increasing pH presented a positive effect on surface tension and emulsion stability, according to Abouseud et al. [70], no significant increase in BS and BE activity were observed for any strains; only *B*. *vallismortis* in sessile culture showed the highest EI_24_ (76.6%) under the condition of pH 8 at 10 rpm and with 4.5% NaCl (CC8), surpassing the results found in the other assays (Figure 4a). In *B*. *siamensis* and *B*. *vallismortis* no effect was observed with the addition of salts, while *Paenibacillus* sp. and *Bacillus* sp. have a lower percentage of EI_24_, with statistically significant differences (*p* < 0.05) between the different salt concentrations (Figure 4a). In a planktonic culture (Figure 4b), only *B*. *siamensis* recorded BE activity in all conditions where agitation was equal to or greater than 150 rpm. Although an increase in drop-collapse and EI_24_ was not observed with agitation, it was observed that high agitation values kept BE activity stable (Figure 4b), regardless of salinity and pH, and it has been reported that high agitation ranges can improve the production of BS and BE [71].

Regarding collapsed drop, results similar to those found in the production of different times and the optimization of the medium were observed in both sessile (Figure 5a) and planktonic (Figure 5b) cultures; the strains of the genus *Bacillus* (XHA14, XHA16 and TZS01) showed BS activity ≥4 mm. However, this droplet size fluctuated in different culture conditions, but it usually did not decrease below 5 mm. The ability of the *Bacillus* genus to produce BS compounds is well known [23,72], where molecular studies of the non-ribosomal peptide synthetase (NRPS) group confirm the chemical nature of a BS belonging to surfactins [73]. In both sessile and planktonic cultures, *Paenibacillus* sp. and *L. fusiformis* did not present significant BS activity (≥4 mm) under any culture condition (Figure 5a,b), consistent with the results found in the BS and BE expression at different times, as well as in the optimization of the medium. The importance of sessile culture in the optimization should be highlighted; except *L. fusiformis*, the rest of the bacteria maintain a BE activity under the different salt and pH conditions. Finally, to our knowledge, this is the first study where the optimization of the C/N source and the sessile culture conditions for the expression of BS and BE type metabolites was carried out.

## 4. Conclusions

Sinkholes represent a great opportunity for the bioprospection of microorganisms that produce compounds with emulsifying and surfactant activity that are stable over time and tolerate different salinity conditions for possible biotechnological, industrial and commercial applications.

Eighty strains with droplet size ≥4 mm (BS activity) and EI_24_ ≥ 50% (BE activity) were obtained, of which seventy-seven were considered halotolerant; therefore, in salinity conditions of 5% and 10%, 42 with BE activity and 24 BS activity were stable. Haemolysis results show that strains with BS activity do not necessarily have haemolytic activity.

In the molecular identification, the genera with the highest BS and BE activity were *Bacillus*, *Paenibacillus* and *Lysinibacillus*. The time of greater expression of the BS and BE compounds fluctuated depending on the species; however, *B. vallismortis* and *Bacillus* sp. maintained their BE activity at different hours, while BS activity was stable for *B. vallismortis*, the latter also showed BS and BE activity in different salinity conditions. In optimising the culture medium to evaluate the expression of BS and BE activity, there are no notable significant differences with the commercial culture medium, although a better BS activity was observed in planktonic culture. This study highlights the relevance of the economic substrates used in the tests with promising results for obtaining compounds of interest. It was found that the nitrogen source positively affects BS and BE activity regardless of the culture type. However, in the sessile culture conditions a similar BS activity was observed at the different times evaluated but not in planktonic cultures.

These results conclude that biosurfactants and bioemulsifiers produced by bacteria isolated from sinkholes could have greater advantages in remediation processes than their synthetic counterparts, which form toxic by-products. It should be noted that the metabolites responsible for the BS and BE activity were not purified; even so, they reached values equal to or higher than the purified compounds. Therefore, these strains have high biotechnological potential; however, it is necessary to carry out more tests and evaluation criteria and ensure the purification of the compounds responsible for the BS and BE activities, and in vitro and in situ degradation tests of hydrocarbons or contaminated substrates.

## Figures and Tables

**Table 1:** Media composition (C/N source) and culture conditions for the BS and BE production of bacteria isolated from sinkholes.

	C/N Source Concentration								
Composition (%)	CN1	CN2	CN3	CN4	CN5	CN6	CN7	CN8	CN9
Molasses	1	1	1	2	2	2	3	3	3
Yeast extract	0.55	1.25	2	0.55	1.25	2	0.55	1.25	2
	**Culture Conditions**								
Culture condition	CC1	CC2	CC3	CC4	CC5	CC6	CC7	CC8	CC9
NaCl (%)	0.5	0.5	4.5	4.5	0.5	0.5	4.5	4.5	2.5
pH	6	8	6	8	6	8	6	8	7
Planktonic agitation (rpm)	180	180	180	180	120	120	120	120	150
Sessile agitation (rpm)	4	4	4	4	10	10	10	10	7

**Table 2:** Taxonomy of bacteria strains from two pristine sinkholes of the Yucatan Peninsula selected for their BS and BE activity stable under salinity conditions, NaCl tolerance and haemolytic activity.

Identification					Emulsification Index (%)			Drop-Collapse (mm)			Haemolytic Activity
Strain	Similarity (%)	GenBank Accessions	Nearest Phylogenetic Neighbour	Tolerance NaCl (%)	TSB	NaCl 5%	NaCl 10%	TSB	NaCl 5%	NaCl 10%	CFS (mm)
TZA46	97.73	ON600577	*Brevibacillus* sp.	2.5	65.51	63.62	57.40	3.0	3.0	3.0	1.8
XHA18	98.37	ON600579	*Paenibacillus* sp. *	7.5	65.15	61.59	61.24	3.5	4.9	4.2	(−)
TZA38	99.18	ON600589	*Lysinibacillus fusiformis* *	5.0	65.10	63.20	60.33	4.1	3.1	3.4	(−)
TZA34	94.00	ON586647	*Pseudomonas aeruginosa*	7.5	64.38	64.43	55.79	3.0	4.0	4.0	2.1
TZA10	88.00	ON600528	*Pseudomonas* sp.	10.0	64.38	53.79	50.95	3.0	3.0	3.0	1.7
XHA90	97.46	ON600581	*Bacillus* sp.	20.0	63.94	62.22	62.04	3.0	4.0	3.0	(−)
XHA66	96.84	ON606036	*Cytobacillus* sp.	10.0	63.57	62.89	61.16	3.0	3.0	3.0	(−)
XHA28	88.60	ON600618	Family Bacillaceae	17.5	63.11	62.07	38.16	3.0	3.0	3.0	(−)
TZA26	98.63	ON600536	*Staphylococcus epidermidis*	15.0	61.69	55.82	6.32	4.1	3.0	3.0	(−)
TZA51	99.32	ON595357	*Lysinibacillus fusiformis*	5.0	61.61	61.91	58.63	3.0	3.0	3.0	0.2
TZRP2	98.83	ON600664	*Serratia rubidaea*	2.5	61.58	54.95	51.80	3.0	4.0	4.0	(−)
TZA11	99.59	ON600582	*Lysinibacillus sphaericus*	12.5	60.29	54.53	53.79	3.0	3.0	3.0	(−)
XHA53	98.77	ON599339	*Staphylococcus epidermidis*	10.0	59.66	53.68	53.32	4.5	4.0	3.0	1.8
XHA78	98.83	ON600578	*Pseudomonas parafulva*	5.0	59.60	41.27	36.89	3.0	3.0	3.0	(−)
XHA33	98.60	ON600531	*Staphylococcus* sp.	15.0	59.50	58.10	41.67	3.5	4.0	4.0	1.5
TZA50	90.70	ON606021	Family Rhizobiaceae	2.5	58.27	58.24	54.96	4.9	4.0	4.0	(−)
TZS01	98.17	ON599903	*Bacillus* sp. *	12.5	57.80	53.36	55.58	5.9	6.7	6.7	1.2
XHA06	98.27	ON600721	*Pseudomonas* sp.	12.5	57.73	60.94	54.95	6.2	5.3	4.7	1.7
XHA16	98.77	ON600576	Bacillus vallismortis *	12.5	57.73	60.94	54.95	7	7.8	7.2	1.7
XHA14	99.11	ON600533	Bacillus siamensis *	12.5	57.63	57.03	53.71	6.7	7.3	7.2	2
TZA04	98.84	ON600529	*Bacillus amyloliquefaciens*	12.5	56.11	54.95	52.83	5.6	4.0	4.0	1.6
TZA15	82.30	ON606020	Family Sphingobacteriaceae	7.5	55.95	64.37	44.94	3.0	4.0	4.5	(−)
TZA47	95.69	ON606022	*Brevibacillus* sp.	2.5	53.02	49.87	41.35	3.8	5.0	5.3	(−)
XHA01	98.90	ON600876	*Pseudomonas aeruginosa*	7.5	3.29	59.91	60.07	4.4	6.0	6.0	2.2

* Strains selected for the BS and BE production trials at different times, optimization of the medium, and culture conditions; (−) No haemolytic activity.

## Data Availability

Not applicable.

## Supplementary Materials

The following supporting information can be downloaded at: https://www.mdpi.com/article/10.3390/microorganisms10071264/s1, Figure S1. Morphology of some strains isolated from sinkholes (a) and NaCl tolerance gradient (b), Figure S2. Evolutionary relationships of taxa. The evolutionary history was inferred using the Neighbour-Joining method. The bootstrap consensus tree inferred from 1000 replicates. Evolutionary analyses were conducted in MEGA X, Figure S3. Response surface to the bioemulsifier of bacteria isolated from sinkholes by different concentrations of C/N in sessile and planktonic culture, Figure S4. Response surface to the biosurfactant activity of bacteria isolated from sinkholes by different concentrations of C/N in sessile and planktonic culture. Table S1. Biosurfactant (drop-collapse ≥ 4 mm) and/or bioemulsifier (EI_24_ 50%) activity stable at different salt concentrations, tolerance to NaCl, pH and haemolytic activity of bacteria isolated from the X′ can ho che (XH) and Temozón (TZ) sinkholes, Table S2. Principal component analysis (PCA) correlations of the collapsed droplet and emulsification index under different combinations of C/N source, in sessile and planktonic bacterial culture conditions.
