# Supplementary material for: Bacteria Halotolerant from Karst Sinkholes as a Source of Biosurfactants and Bioemulsifiers
Source: Microorganisms. 2022 Jun 21;10(7):1264. doi: 10.3390/microorganisms10071264 (PMC9319531; doi:10.3390/microorganisms10071264)
Supplement: Supplementary file 1 [file microorganisms-10-01264-s001.zip › Table S1.pdf]

Table S1. Biosurfactant (drop collasep ≥4 mm) and/or bioemulsifier (EI_{24} ≥50%) activity stable at different salt concentrations, tolerance to NaCl, pH and haemolytic activity of bacteria isolated from the X´can ho che (XH) and Temozón (TZ) sinkholes.

	Strain	Specie as close relatives	Emulsification index EI_{24} (%)			Drop collapse (mm)			Tolerance a NaCl (%)	pH	Haemolytic activity (mm)
			TSB	NaCl 5%	NaCl 10%	TSB	NaCl 5%	NaCl 10%			
1	XHA01	*Pseudomonas aeruginosa*	3.29	59.91	60.07	4.44	6.00	6.00	7.5	7.80	2.2
2	XHA03	*Burkholderia cepacia*	52.48	0.00	0.00	3.00	3.00	3.00	10.0	7.80	(-)
3	XHA06	*Pseudomonas* sp.	57.75	35.39	12.92	3.00	3.00	3.00	0.5	8.40	(-)
4	XHA10	Bacillus Gram negative	57.57	16.14	13.23	4.75	5.00	5.00	17.5	8.40	2
5	XHA13	Bacillus Gram negative	52.89	14.35	11.38	4.00	3.00	3.00	7.5	8.00	(-)
6	XHA14	*Bacillus siamensis*	39.17	60.84	55.96	6.71	6.19	6.13	12.5	7.90	2
7	XHA15	Bacillus Gram negative	21.95	0.73	0.50	4.31	3.00	3.00	10.0	8.20	1.6
8	XHA16	*Bacillus vallismortis*	57.73	60.94	54.95	6.15	5.31	4.65	12.5	7.60	1.7
9	XHA18	*Paenibacillus* sp.	65.15	61.24	62.29	3.00	3.00	3.00	7.5	7.80	(-)
10	XHA19	Bacillus Gram negative	2.26	0.00	0.00	6.69	6.52	6.30	17.5	8.00	2
11	XHA22	Bacillus Gram positive	45.45	27.85	22.63	4.00	3.00	3.00	10.0	8.00	(-)
12	XHA24	Bacillus Gram negative	35.26	22.78	19.86	4.36	4.00	4.00	12.5	7.30	1.9
13	XHA25	*Stenotrophomonas* sp.	33.64	0.00	0.00	4.19	3.00	3.00	10.0	7.60	1.7
14	XHA28	Family Bacillaceae	63.11	62.07	38.16	3.00	3.00	3.00	17.5	8.10	(-)
15	XHA30	Coccus Gram positive	55.85	9.92	6.60	3.00	4.00	3.00	15.0	8.60	1.1
16	XHA33	*Staphylococcus* sp.	59.50	58.10	41.67	3.50	4.00	4.00	17.5	6.70	1.5
17	XHA36	Coccus Gram positive	58.30	0.00	0.00	3.00	3.00	3.00	20.0	8.20	1.4
18	XHA38	*Photobacterium* sp.	50.12	0.00	0.00	3.00	3.00	3.00	20.0	8.40	(-)
19	XHA40	Bacillus Gram positive	52.80	0.00	0.00	3.00	3.00	3.00	5.0	8.20	(-)
20	XHA42	*Burkholderia cepacia*	56.18	0.00	0.00	3.00	3.00	3.00	20.0	7.90	(-)
21	XHA46	Bacillus Gram negative	19.92	0.00	0.00	4.00	3.00	3.00	10.0	8.30	(-)
22	XHA47	Bacillus Gram negative	54.95	0.00	0.00	3.00	3.00	3.00	5.0	8.00	(-)
23	XHA52	Bacillus Gram negative	2.90	0.00	0.00	4.00	3.00	3.00	10.0	8.20	(-)
24	XHA53	*Staphylococcus epidermidis*	59.66	53.68	53.32	4.50	4.00	3.00	10.0	8.10	1.8
25	XHA58	Bacillus Gram negative	50.56	0.00	0.00	3.00	3.00	3.00	17.5	8.00	1.8
26	XHA60	Coccus Gram positive	54.30	18.85	14.42	3.00	4.63	4.63	20.0	8.10	(-)
27	XHA61	Bacillus Gram -	48.38	0.10	0.10	4.67	4.00	3.00	10.0	7.90	2.1
28	XHA62	Bacillus Gram positive	54.19	0.00	0.00	4.00	4.00	3.50	10.0	7.90	2.2
29	XHA66	*Cytobacillus*. sp.	63.57	62.89	61.16	3.00	3.00	3.00	10.0	6.10	(-)
30	XHA69	Bacillus Gram positive	51.56	0.00	0.00	3.00	3.00	3.00	7.5	8.10	2.1
31	XHA70	Bacillus Gram negative	56.11	0.00	0.00	3.00	3.00	3.00	5.0	8.00	(-)
32	XHA72	Bacillus Gram negative	51.51	0.00	0.00	3.00	3.00	3.00	10.0	7.60	1.9
33	XHA74	Diplococci Gram positive	55.70	49.73	34.03	3.00	4.38	4.44	7.5	8.30	2
34	XHA76	Coccus Gram negative	54.14	43.19	19.55	3.00	3.00	3.00	12.5	7.80	(-)
35	XHA78	*Pseudomonas parafulva*	59.60	41.27	36.89	3.00	3.00	3.00	5.0	7.00	(-)
36	XHA80	Bacillus Gram negative	38.52	0.00	0.00	3.50	3.00	3.00	10.0	8.10	(-)
37	XHA84	Cocobacillus Gram negative	12.03	11.71	3.26	4.13	3.00	3.00	17.5	8.30	(-)
38	XHA85	Bacillus Gram positive	51.85	0.00	0.00	3.00	3.00	3.00	10.0	7.60	(-)
39	XHA88	Bacillus Gram negative	4.84	0.00	0.00	4.00	3.00	3.00	12.5	8.00	2
40	XHA90	*Bacillus* sp.	63.94	62.22	62.04	3.00	4.00	3.00	20.0	7.40	(-)
41	XHA91	Bacillus Gram negative	53.20	0.00	0.00	3.00	3.00	3.00	5.0	7.90	(-)
42	XHA96	Bacillus Gram negative	53.97	37.19	30.77	4.00	6.00	5.00	10.0	7.50	2.1
43	XHA98	Bacillus Gram positive	43.92	37.59	28.00	5.00	3.00	3.00	10.0	7.30	1.9
44	XHA99	Bacillus Gram negative	0.24	0.00	0.00	4.00	3.00	3.00	5.0	7.90	(-)
45	XHA102	Bacillus Gram negative	46.00	0.00	0.00	4.00	3.00	3.00	10.0	7.90	(-)
46	XHA104	Bacillus Gram negative	57.82	0.00	0.00	3.63	3.00	3.00	0.5	7.70	1.9
47	XHA105	Bacillus Gram negative	56.91	56.87	34.35	3.75	3.00	3.00	2.5	7.80	(-)
48	XHA109	Bacillus Gram negative	55.30	0.00	0.00	3.00	3.00	3.00	7.5	7.20	1.9
49	TZA01	*Pseudomonas luteola*	20.41	0.00	0.00	5.88	5.00	4.00	12.5	7.80	1.9
50	TZA02	Bacillus Gram negative	48.77	0.00	0.00	5.69	4.00	4.00	17.5	7.90	1.9
51	TZA03	*Pseudomonas luteola*	3.44	28.70	0.28	6.56	6.38	5.56	10.0	7.60	1.7
52	TZA04	*Bacillus amyloliquefaciens*	56.11	54.95	52.83	5.63	4.00	4.00	12.5	7.60	1.6
53	TZA05	*Bacillus* sp.	56.88	51.06	48.00	6.75	6.53	6.38	12.5	7.80	1.8

54	TZA07	*Pseudomonas luteola*	0.95	62.01	54.51	5.69	4.00	4.00	12.5	7.60	1.9
55	TZA08	Bacillus Gram -	0.83	0.00	0.00	5.44	5.56	5.06	12.5	7.70	2
56	TZA10	*Pseudomonas* sp.	64.38	53.79	50.95	3.00	3.00	3.00	10.0	7.90	1.7
57	TZA11	*Lysinibacillus sphaericus*	60.29	54.53	53.79	3.00	3.00	3.00	12.5	7.70	(-)
58	TZA13	*Pseudomonas luteola*	60.31	4.77	3.74	3.00	3.00	3.00	15.0	7.70	1.6
59	TZA15	Family Sphingobacteriaceae	55.95	64.37	44.94	3.00	4.00	4.50	7.5	7.70	(-)
60	TZA17	Bacillus Gram positive	53.36	29.72	28.80	3.00	4.75	4.75	12.5	7.60	1.5
61	TZA18	Bacillus Gram negative	52.54	41.62	37.81	3.00	3.00	3.00	10.0	7.60	1.4
62	TZA21	Coccus Gram positive	58.66	5.54	5.49	3.00	3.00	3.00	2.5	8.00	(-)
63	TZA26	*Staphylococcus epidermidis*	61.69	55.82	6.32	4.06	3.00	3.00	15.0	8.20	(-)
64	TZA27	Coccus Gram negative	56.38	0.00	0.00	3.00	3.00	3.00	5.0	7.70	(-)
65	TZA32	Coccus Gram positive	49.69	35.39	12.92	4.56	3.00	3.00	5.0	7.90	2
66	TZA34	*Pseudomonas aeruginosa*	64.38	64.43	55.79	3.00	4.00	4.00	7.5	7.30	2.1
67	TZA37	Coccobacillus Gram -	59.49	2.63	4.09	3.00	3.00	3.00	10.0	8.20	(-)
68	TZA38	*Lysinibacillus fusiformis*	65.10	63.20	60.33	3.00	3.00	3.00	5.0	8.00	(-)
69	TZA44	Coccus Gram negative	54.25	0.00	0.00	4.56	4.00	4.00	7.5	8.10	(-)
70	TZA45	Bacillus Gram positive	63.07	47.30	41.14	5.31	4.25	4.00	17.5	7.80	1.3
71	TZA46	*Brevibacillus* sp.	65.51	63.62	57.40	3.00	3.00	3.00	2.5	8.10	1.8
72	TZA47	*Brevibacillus* sp.	53.02	49.87	41.35	3.78	5.00	5.25	2.5	7.30	(-)
73	TZA50	Familia Rhizobiaceae	58.27	58.24	54.96	4.86	4.00	4.00	2.5	8.30	(-)
74	TZA51	*Lysinibacillus fusiformis*	61.61	61.91	58.63	3.00	3.00	3.00	5.0	9.00	(-)
75	TZA53	*Burkholderia cepacia*	6.44	0.00	0.00	4.25	4.00	3.00	7.5	7.70	1.7
76	TZRP2	*Serratia rubidaea*	61.58	54.95	51.80	3.00	4.00	4.00	2.5	7.80	(-)
77	TZRP3	*Burkholderia cepacia*	50.95	39.42	40.05	3.00	3.00	3.00	15.0	7.90	2.1
78	TZRP5	*Bacillus cereus* var. *cereus*	57.92	0.00	0.00	5.14	6.00	5.00	5.0	7.50	(-)
79	TZS01	*Bacillus* sp.	57.80	53.36	54.26	4.63	4.00	4.00	12.5	7.60	1.2
80	TZS02	Bacillus Gram negative	56.95	55.53	55.45	4.88	3.00	3.00	12.5	7.60	1.7
