# Supplementary material for: Bacteria Halotolerant from Karst Sinkholes as a Source of Biosurfactants and Bioemulsifiers
Source: Microorganisms. 2022 Jun 21;10(7):1264. doi: 10.3390/microorganisms10071264 (PMC9319531; doi:10.3390/microorganisms10071264)
Supplement: Supplementary file 1 [file microorganisms-10-01264-s001.zip › Table S2.pdf]

Table S2. Principal component analysis (PCA) correlations of the collapsed droplet and emulsification index under different combinations of C/N source, in sessile (S) and planktonic (P) bacterial culture.

	Drop collapse (mm)		Emulsification index (EI_{24})	
Strains	Nitrogen source	Carbon source	Nitrogen source	Carbon source
Bacillus siamensis XHA14-P	-0.313690	-0.504833	0.437903	-0.433013
Bacillus vallismortis XHA16-P	0.529656	-0.146911	0.546260	-0.621689
Paenibacillus sp. XHA18-P	-0.140902	0.456522	0.821356	0.334431
Bacillus sp. TZS01-P	0.244418	-0.697329	0.306472	-0.625970
Lysinibacillus fusiformis TZA38-P	0.000936	-0.787532	0.727082	-0.152237
Bacillus siamensis XHA14-S	0.584840	-0.188207	0.752091	-0.091984
Bacillus vallismortis XHA16-S	-0.274070	-0.699446	0.724748	-0.006858
Paenibacillus sp. XHA18-S	0.561925	-0.177646	0.660008	0.570003
Bacillus sp. TZS01-S	0.648176	-0.379049	0.437563	-0.443241
Lysinibacillus fusiformis TZA38-S	0.029721	0.266228	-0.426036	0.563514
