# Supplementary figures and images for: Bacteria Halotolerant from Karst Sinkholes as a Source of Biosurfactants and Bioemulsifiers
Source: Microorganisms. 2022 Jun 21;10(7):1264. doi: 10.3390/microorganisms10071264 (PMC9319531; doi:10.3390/microorganisms10071264)

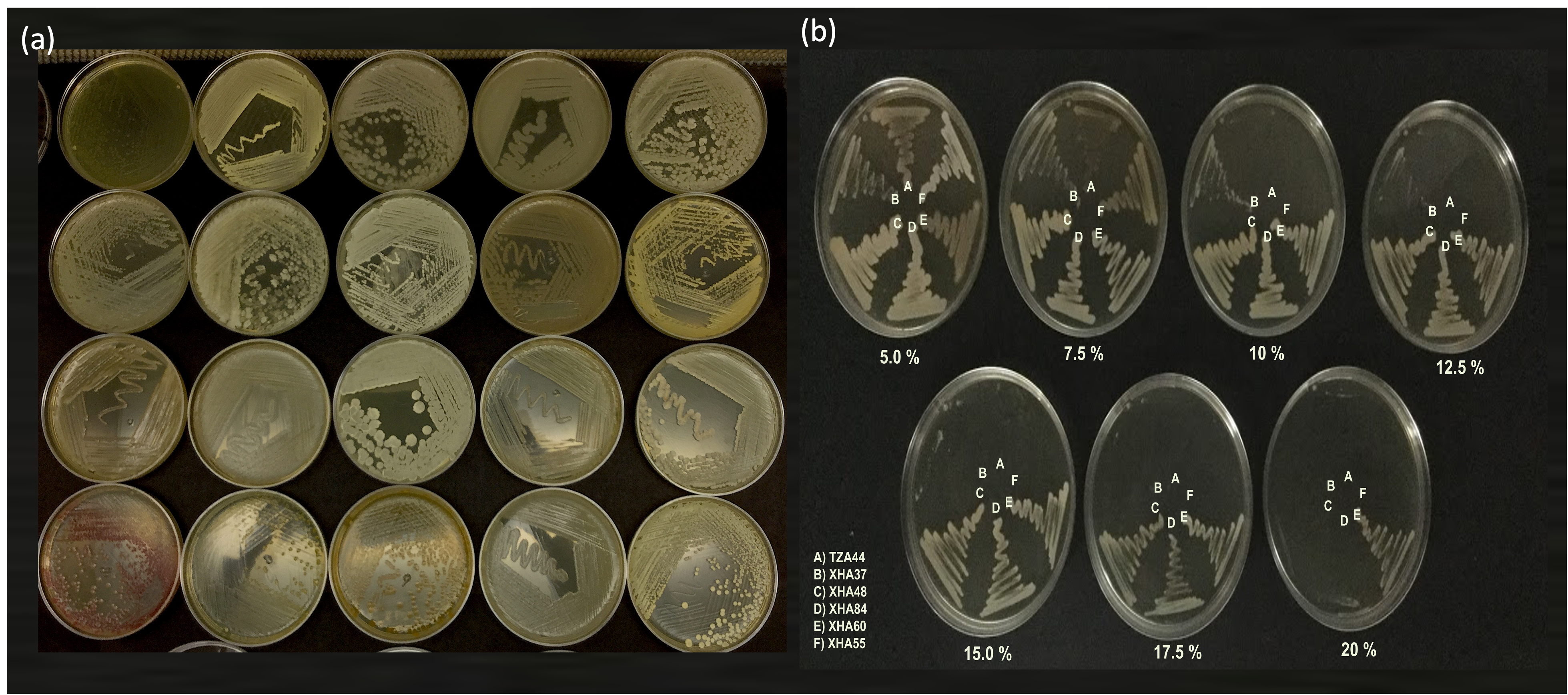

Supplement: Supplementary file 1 [file microorganisms-10-01264-s001.zip › Figure S1.png]

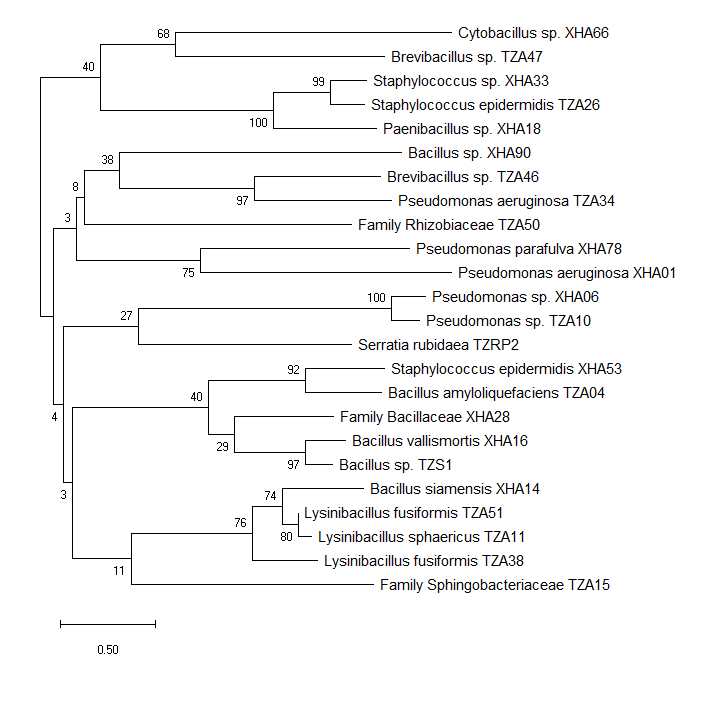

Supplement: Supplementary file 1 [file microorganisms-10-01264-s001.zip › Figure S2.png]

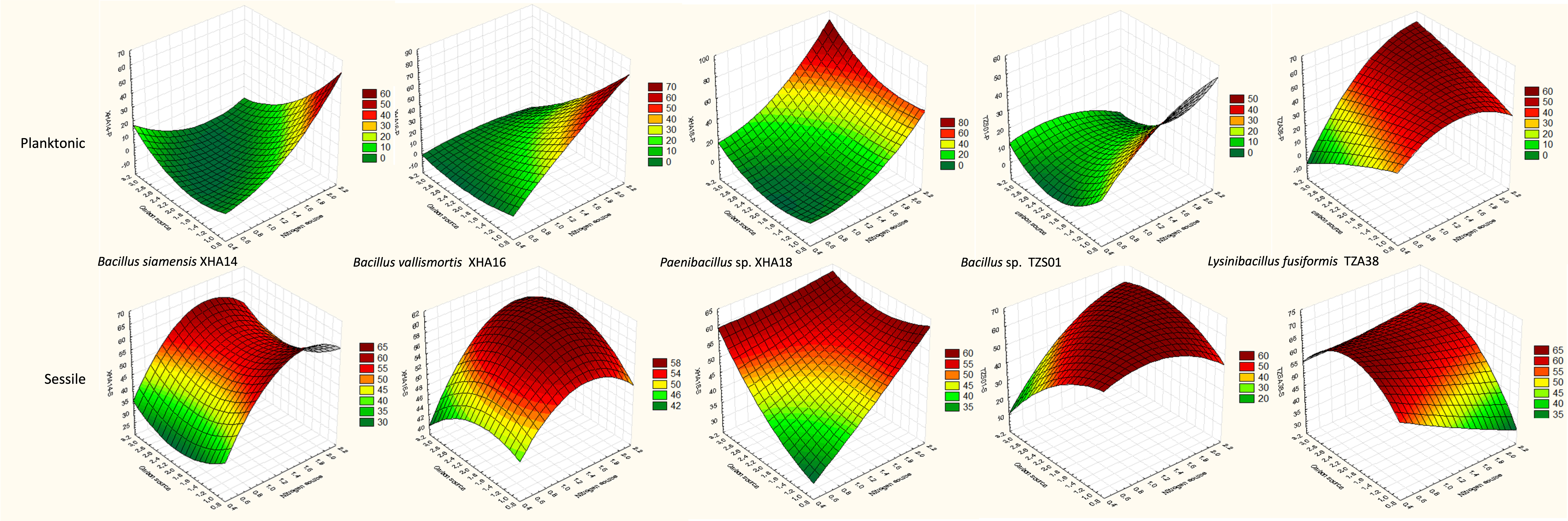

Supplement: Supplementary file 1 [file microorganisms-10-01264-s001.zip › Figure S3.png]

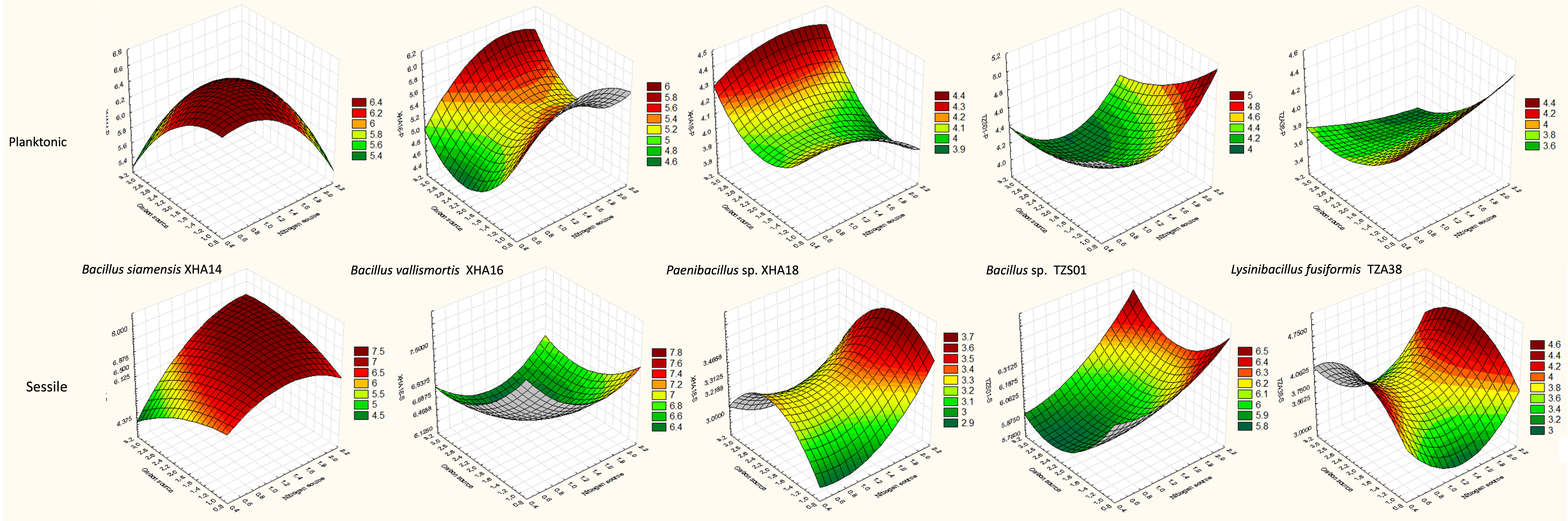

Supplement: Supplementary file 1 [file microorganisms-10-01264-s001.zip › Figure S4.png]
